# Actual practices of community pharmacists in the management of diabetes: a comparison of simulated patient-based study with perceived role of involvement

**DOI:** 10.1080/20523211.2024.2326381

**Published:** 2024-03-28

**Authors:** Ashenafi Kibret Sendekie, Amensisa Hailu Tesfaye, Yabibal Berie Tadesse, Abera Dessie Dagnaw, Eyayaw Ashete Belachew

**Affiliations:** a Department of Clinical Pharmacy, School of Pharmacy, College of Medicine and Health Sciences, University of Gondar, Gondar, Ethiopia; b Department of Environmental and Occupational Health and Safety, Institute of Public Health, College of Medicine and Health Sciences, University of Gondar, Gondar, Ethiopia; c Department of Pharmaceutical Chemistry, School of Pharmacy, College of Medicine and Health Sciences, University of Gondar, Gondar, Ethiopia

**Keywords:** Actual practices, Community pharmacists, Diabetes, Perceived involvement, Northwest Ethiopia, Simulated patients

## Abstract

**Objectives:**

This study evaluated the level of community pharmacy professionals’ (CPPs) actual practices and contrasted it with their self-reported perceived involvement in diabetes management.

**Methods:**

A self-reported cross-sectional and simulated patient (SP)-based study were employed at community drug retail outlets (CDROs) in Northwest Ethiopia. SP-case scenarios were used to examine the actual practices of CPPs in diabetes management and were compared with self-reported perceived involvement. The data were managed and analysed using SPSS version 26.

**Results:**

About 184 participants in the self-reported and 100 CPPs’ actual practices using three SP visits were included. The overall actual practice (17.8%) was found to be significantly different compared with the perceived level of involvement (73.5%) (*p*-value <0.05). About 94.3% of CPPs dispensed requested medications to the SP without a prescription. Despite most participants being perceived to be involved, more than 76% of CPPs did not counsel the SP for lifestyle modifications, avoiding risky behaviours, adherence to treatment, routine blood glucose checkups, diabetic foot care techniques, or consultation with physicians for further management.

**Conclusion:**

A significant discrepancy between actual practices and perceived CPPs’ involvement in the management of diabetes was observed. The findings may suggest that exploring possible gaps may be crucial.

## Introduction

The roles of community pharmacist in public healthcare services have grown and expanded. They are making more direct interventions in disease management, medication education, and preventative actions (Omboni & Caserini, 2018; George et al., 2010; Sendekie et al., 2023; Laliberté et al., 2012). Direct interventions can be used to provide people in the area with pharmaceutical treatments immediately. This may lead to increased medication adherence, desired therapeutic results, and safer medication usage procedures (Omboni & Caserini, 2018; George et al., 2010; Ali et al., 2012).

Diabetes continues to be a major public health concern, with expected cases of 783 million diabetes worldwide by 2045 (Sun et al., 2022). Three-fourths of patients with diabetes are located in low-and- middle income countries (LMICs) (Ogurtsova et al., 2017). Additionally, its burden has increased, particularly in LMICs, highlighting the critical need for diabetes control strategies (Islam et al., 2014; World Health Organization World Health Statistics, 2015). Furthermore, with 24 million cases expected to be detected up until 2021 and an estimated 55 million by 2045, diabetes has been considered as one of the major public health risks in Africa (Sun et al., 2022). More than 2.5 million adults in Ethiopia are also affected (International Diabetes Federation, 2017), and the projected prevalence increased sharply from 3.8% in 2016 to 5.2% in 2017 (Bishu et al., 2019). Many factors associated with diabetes are closely related to sedentary lifestyles, including obesity, physical inactivity, a poor diet, and smoking. Chronic diseases such as dyslipidemia, diabetes, cardiovascular diseases, renal disorders, and retinopathy may develop as a result of these risk factors (Demilew & Firew, 2019; Awad & Al-Nafisi, 2014). Age, alcohol use, and abdominal obesity have all been noted as major risk factors for emerging chronic diseases in Ethiopia (Ali et al., 2021; Aragaw et al., 2020). Therefore, controlling modifiable risk factors and changing sedentary lifestyle patterns can help lower these risks (Lavie et al., 2019). Also, effective risk factor management has been implemented to lower mortality and morbidity risks and enhance healthcare systems for both chronic diseases with and without a diagnosis (De Backer et al., 2004).

Community drug retail outlets (CDROs) are the initial healthcare sectors where patients can access medications and treatments. In a number of healthcare services, community pharmacy professionals (CPPs) are crucial [ Nsengimana et al., 2022; Erku et al., 2017; Gelayee & Mekonnen, 2017; Mensah et al., 2018; Offu et al., 2015; Yimer et al., 2020). Additionally, they can aid in the management and prevention of chronic conditions like metabolic syndromes, diabetes, and cardiovascular diseases, which will improve patient care (George et al., 2010; Erku et al., 2017; Belachew et al., 2020; Yousuf et al., 2019; Dhippayom et al., 2013; Katangwe et al., 2019; Malik et al., 2022). It was also discovered that a diabetic intervention provided by CPPs had better results in treatment outcome (Ali et al., 2012; Malik et al., 2022; Farag Mohamed et al., 2021; Al Assaf et al., 2022).

Studies in Ethiopia have also looked into the role of CPPs in the prevention and management of diabetes (Erku et al., 2017; Belachew et al., 2020; Teka & Baye, 2018; Sendekie et al., 2023). All these findings are self-reported perceived levels of involvement and reflect how CPPs perceive their own role in preventing and managing diabetes. However, evidence is still scarce, especially when it comes to actual practice. The existing literature focuses primarily on self-reported findings. It might be challenging to generalise about the actual levels of CPPs’ involvement based on self-reported findings. The scarcity of evidence on the actual practice of CPPs’ involvement in diabetes management reflects the need for more research in this area. This is important because CPPs are well-positioned to play a significant role in diabetes management, given their accessibility and expertise in medication management. In order to analyse actual practices, this current study compared it to a self-reported cross-sectional survey that had considerably more study participants and was conducted among the three largest cities in the country. The self-reported cross-sectional study that evaluated the perceived level of involvement as the first component of this project was already published (Sendekie et al., 2023). As a result, this study examined the actual counseling practice of CPPs and compared it to a self-reported perceived level of involvement in the management of diabetes at selected CDROs in Northwest Ethiopia.

## Methods and materials

### Study design, settings, and samples

This study was conducted using a self-reported cross-sensational and SP-based scenario of CPPs employed by CDROs in Northwestern Ethiopia. The actual practice of CPPs in the management of diabetes was assessed using a SP-based study and then compared with the self-reported cross-sectional study, which was used to assess the perceived levels of practices of CPPs in managing diabetes. The two phases were employed consecutively, and the first was a self-reported study that was conducted between September 1 and 30, 2022. From October 15 to November 30, three SP visits were conducted as part of the SP-based study, which was conducted in CDROs that took part in the self-reported survey. Firstly, the study areas, Debre Tabor, Bahira Dar, and Gondar cities were selected using lottery method randomly from the other cities in the area.

In the self-reported cross-sectional study, pharmacists included in the study were selected from active CDROs located in these three cities. Considering the small number of licensed CDROs available in the selected cities and our lack of the total number of CPPs per CDRO, we proposed contacting one participant per CDRO. Then, we got in touch with every CDRO that was still in operation at the time the data was being collected. We randomly interviewed one CPP volunteer if there were many CPPs per CDRO. CPPs who refused to participate in the research or were not reachable at the time of data collection were not included. Finaly, the self-reported cross-sectional survey included 184 respondents. CDROs, we approached in the SP-based study were similar to those in the self-reported cross-sectional study, and those who were included in the self-reported cross-sectional study were considered in the SP-based study. CPPs who were not working during any SP visits and those who reported that any requested medications were not available and who were unable to provide the service were excluded from the analysis. Out of 184 CPPs approached, we assessed the actual practice of 100 CPPs using three SP visits to assess their actual practice in diabetes management.

### Definition of terms

**Actual practices:** In this study, it refers to real-world engagement, interactions, and service provision towards managing a patient with diabetes by CPPs. The actual practice was determined using practice items that CPPs were expected to address while managing a patient with diabetes.

**Community pharmacy professionals:** In this study, it refers to pharmacy professionals who worked at CDROs in the selected cities, regardless of their educational background. In terms of educational background, they might be either diploma holders (lower educational background) or those with a bachelor degree and above (higher educational background).

**Perceived involvement:** It shows how much CPPs believe they are involved in the treatment of diabetes in terms of participation and engagement in promotion, counseling, education, and service provision. The level of perceived involvement was determined using CPPs’ perception on items that pharmacists were expect to address in managing patients with diabetes. These items were on a Likert scale with five levels (not at all involved, little involved, uncertain, involved, and very involved) (Sendekie et al., 2023).

### Data collection procedures and instruments

Data collection instrument forms for the self-reported cross-sectional and SP-based studies were organised independently. However, the perceived level of CPPs involvement and their actual practices were measured and compared using the same items [**Supplementary file 1**]. These items focus on the routine, detailed activities of CPPs, including: the request for a prescription; detailed medication information in terms of dosage regimens, potential side effects, and adherence; lifestyle modifications and risky behaviours; physical activities; routine checkups for blood glucose, blood pressure, and weight; consulting of physicians for further assessment; and services provided for diabetic patients. The items were prepared after reviewing of relevant literature (Sendekie et al., 2023; Erku et al., 2017; Belachew et al., 2020; Yousuf et al., 2019; Teka & Baye, 2018).

#### Self-reported cross-sectional survey

Data were gathered utilising a self-administered questionnaire. A self-administered questionnaire was given to eligible participants who volunteered to take part in the research. The data collection instrument consisted of sociodemographic characteristics of study participants like sex, age, education levels, work experiences, employment status, and training status. The other section of the data collection tool was items used to measure the perceived level of CPPs’ involvement in managing diabetes. Three clinical pharmacy students collected the data following a half-day training on ethical considerations and data collection methods. The study's objectives were explained to participants before they consented to participate.

Fifteen items on a five-point ordinal scale (not involved, little involved, uncertain, involved, very involved) were used to determine the perceived practice of CPPs in diabetes management. The items could be included in three domains: medication review and counselling (four items), lifestyle and self-care modification-related items (seven items), and diabetes education and clinical intervention (four items). Then, the level of involvement of CPPs for each item was determined as ‘not involved (no = 0)’ if they perceived themselves to be either not at all involved, little involved, or uncertain. Whereas, if they were perceived to be involved or very involved in the provision of these services, their level of involvement was considered ‘involved (yes = 1)’. Consequently, the overall level of perceived involvement was determined using all items from each participant and categorised as low (not involved) for those who perceived themselves to not be involved and high (involved) for those who perceived themselves to be involved. We also calculated the Cronbach's alpha reliability test, which yielded 0.78 and was within the acceptable ranges.

#### Actual counseling practice

Data were gathered using the SP-based scenarios. Five CDROs were given a pretest prior to the SP visits. The pretest was not included in the final computation. The intent of the pretest and the SP approach were explained to each of the five CDROs. The algorithm for the SP and the CP's dialogue was somewhat modified in response to input from the pretest and the participating CDROs. The actual practices of CPPs in diabetes management were subsequently evaluated using the information from the SP visit. The CPP's and SP's conversation was modified using earlier SP algorithms (Sendekie et al., 2023; Erku et al., 2016). After receiving a half-day of instruction on the situations and their communication, three pharmacy students who were graduating and who had volunteered to play certain SP roles applied to the SP-based study. As a result, individuals were acclimated to and used the offered clinical circumstances. The data collectors taught the SPs efficient communication methods. To make sure that the data gathered were coordinated, the case presentations made by the three SPs were also cross-tested. They were told not to divulge or request further information unless absolutely essential in order to verify that the information provided to the SPs was consistent. All SP visitors either provided an empty pharmaceutical container or specifically asked the CPPs for the name of a medication.

### Simulated patients and scenarios

A 30-year-old male was diagnosed with diabetes. In addition, the SP needed to refill the patient’s medications but had no prescription. Furthermore, the SP also had no known comorbidities or drug allergies. The SP made three visits to each CDRO in six weeks, two weeks each, using three different scenarios to fully understand their actual practices.

**In scenario 1**, the SP was responsible for asking about refiling metformin plus NPH insulin.

**Scenario 2** also directed the SP to refill Metformin plus glibenclamide.

**In scenario 3**, the SP was instructed to refill NPH insulin.

The pharmacists in CDROs, however, were required to rule out any additional medical conditions, a history of medication use, and whether the patient had visited doctors or clinics. Additionally, on SP visits, pharmacists were expected to obtain valid prescriptions; otherwise, requested medications would not be filled. Furthermore, pharmacists should advise the SP not to take any additional medications without a prescription. The pharmacists were also expected to inform the patients that they should be monitored and followed up on a regular basis and consult physicians for further treatment.

Using the fifteen practice items that the pharmacist could expect to counsel for a patient with diabetes, the actual practice of CPPs in managing diabetes was categorised as not involved if they did not address the items ‘No  = 0’ and involved if the CPPs addressed these services ‘Yes  = 1.’ The internal reliability of items was examined and found to be acceptable with a Kuder-Richardson Formula 20 (KR-20) of 0.75. The overall level of actual practice was also determined based on the average level of involvement for all items and labelled as involved or not involved. Then the level of overall actual practices and the level of practice for each item were compared with the findings of the level of perceived practice in the self-reported cross-sectional study in managing diabetes.

### Data entry and statistical analysis

The information was gathered, checked for accuracy and cleanliness, and then entered into EPI-Info version 8. After being reviewed for accuracy, precision, and consistency, it was imported into the Statistical Package for Social Science (SPSS) version 26 and analysed. Results were shown as frequencies and percentages for categorical variables. A chi-square test was applied to examine a significant difference (*p*-value < 0.05) between the perceived and actual practices of CPPs in managing diabetes.

## Results

### Sociodemographic characteristics of study participants

A total of 184 CPPs took part in the self-reported cross-sectional study. The majority (53.8%) were male, with a mean age of 32.0 ± 8.1 years. With 4.5 ± 1.6 years of work experience, most of participants (58.2%) possessed a lower educational background (diploma level). Moreover, more than two-thirds of participants (69%) did not receive on-duty training (Table 1).

**Table 1.** Sociodemographic characteristics of study participants, Northwest Ethiopia, 2022 (*N* = 184).

Variables		Frequency (n, %)	Mean (±SD)
Sex	Female	85(46.2)	
	Male	99 (53.8)	
Age in years, mean (± SD)	–	–	32.0(±8.1)
Educational level	Druggist (diploma)	107 (58.2)	
	Bachelor degree and above	77 (41.8)	
Work experience (years)	< 1 year	38 (20.7)	
	1–5 Years	88 (47.8)	4.5(±1.6)
	>5 years	58 (31.5)	
Employment	Owner	37 (20.1)	
	Employee	147 (79.9)	
Monthly income in Ethiopian birr	1500–2999	50 (27.2)	
	3000–4999	89 (48.4)	4625.6(±1721.7)
	≥ 5000	45 (24.4)	
CDRO types	Drug store	91 (49.5)	
	Pharmacy	93 (50.5)	
Clients served per day	<50 50-100 > 100	103 (56)	42.7(±15.3)
		70 (38)	
		11 (6.0)	
Working hours/day:	≤ 8	87 (47.3)	8.8(±3.1)
	> 8	97 (52.7)	
On-job training	Yes	57 (31)	
	No	127 (69)	

### Response to simulated patient requests and perceived involvement of CPPs

Out of 100 participants approached with three visits, a total of 245 (81.7%) responses were included. Unlike self-reported perceived involvement, more than three-fourths (> 76%) of the participants did not actually counsel on salt restriction, smoking cessation, alcohol restriction, adherence to treatment, dosage regimen and possible side effects, exercise and physical activity, consulting physicians for further management, and other items, despite the fact that they were expected to counsel the SP. Surprisingly, about 231 (94.3%) of the CPPs in the selected CDROs did not request a prescription for the SP. Overall, about 82.2% of the participants did not actually address diabetes management services to the request of the SP. The difference in actual practice and perceived level of involvement was also statistically significant in all activities (*p*-value <0.05) (Table 2).

**Table 2.** Responses of pharmacist to simulated patient requests and their perceived involvements.

Practice items pharmacist could expect to counsel for a patient with diabetes:	CPPs’ actual practice (*N* = 245)		CPPs’ perceived practice (*N* = 184)		*p*-value
	No (%)	Yes (%)	high (%)	low (%)	
Salt restriction	198 (80.8)	47 (19.2)	25 (13.6)	159 (86.4)	0.002
Smoking cessation	202 (82.5)	43 (17.5)	26 (14.1)	158 (85.9)	0.01
Alcohol restriction	200 (81.5)	45 (18.5)	29 (15.8)	155 (84.2)	0.01
Adherence to treatment	192 (78.5)	53 (21.5)	29 (15.8)	155 (84.2)	0.003
Dosage regimen and possibles side effects	188 (76.7)	57 (23.3)	30 (16.8)	153 (83.2)	0.023
Exercise and physical activity	188 (76.8)	57 (23.2)	33 (18)	151 (82)	0.012
Routine weight, blood pressure, and blood glucose monitoring	202 (82.4)	43 (17.6)	36 (19.6)	148 (80.4)	0.034
Weight reduction by non-weight bearing diet	210 (85.6)	35 (14.4)	43 (23.9)	140 (76.1)	0.013
Prescription medication	231 (94.3)	14 (5.7)	42 (22.9)	142 (77.1)	0.02
Consumption of cholesterol free-diets	207 (84.6)	38 (15.4)	52 (27.2)	132 (71.8)	0.04
Consumption of vegetables	205 (83.8)	40 (16.2)	58 (31.5)	126 (68.5)	0.035
Involving in measuring weight, blood pressure, and blood glucose	207 (84.4)	38 (15.6)	66 (35.6)	118 (64.4)	0.036
Good foot care techniques	214 (87.5)	31 (12.5)	70 (38.1)	114 (61.9)	0.031
Cautions of over-the-counter drugs or herbal products	205 (83.8)	40 (16.2)	80 (43.5)	104 (56.5)	0.027
Consult physicians for further management	215 (87.6)	30 (12.4)	80 (43.5)	104 (56.5)	0.021
**Overall CPPs’ involvement**	**201** (**82.2)**	**44** (**17.8)**	**49** (**26.5)**	**135** (**73.5)**	**0**.**035**

### CPPs’ actual practices compared with perceived levels of involvement

**Figure 1.**
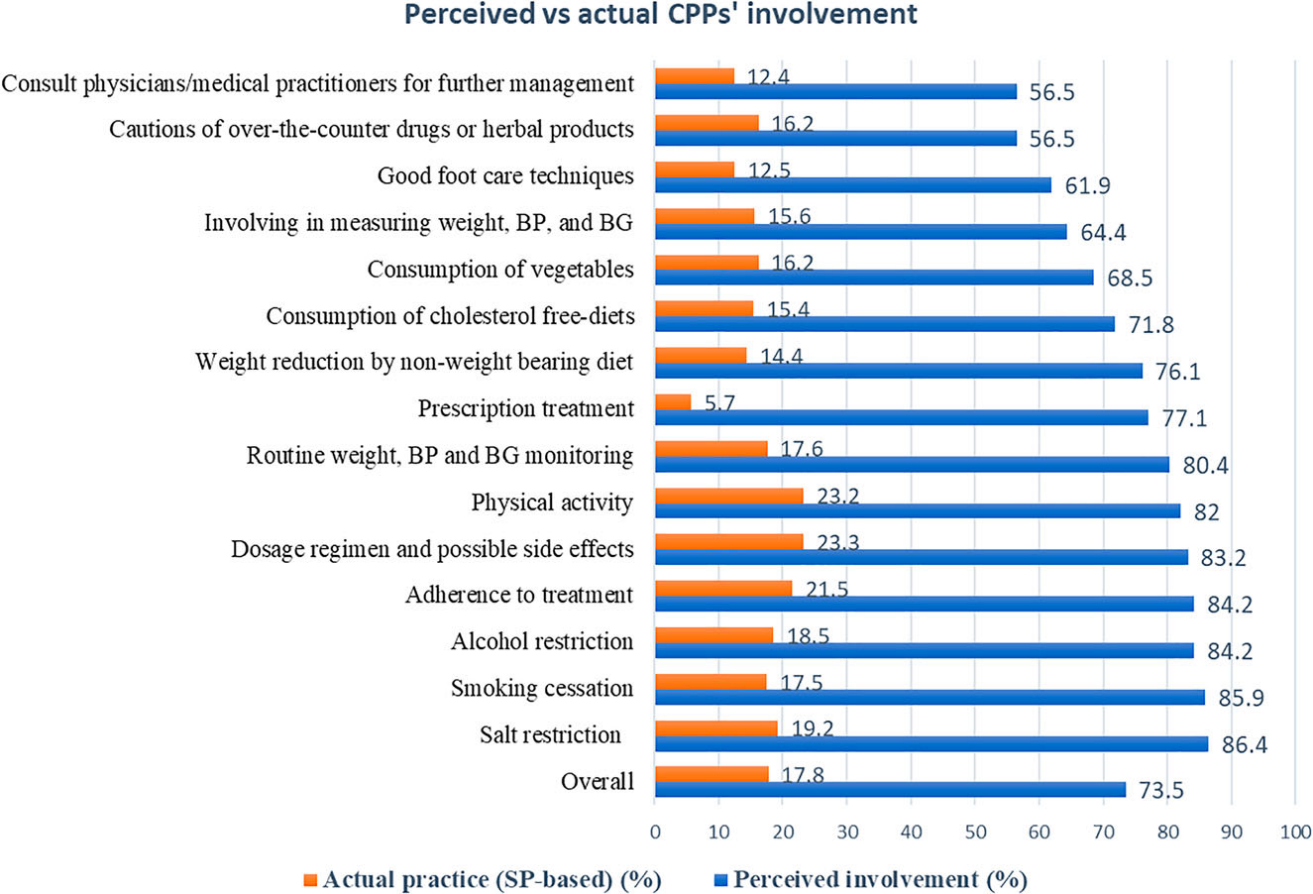
Comparison of perceived level of practices and actual practices of community pharmacists in the management of diabetes.

Contrary to the self-reported perceived level of involvement, more than 76% of the participants did not actually counsel the SP for any services pharmacists were expected to address. Surprisingly, only 5.7% requested SP to provide a prescription, while 77.1% were perceived to counsel prescription-only treatment. Overall, only 17.8% were found to actually address practice items in managing diabetes, despite around three-fourths (73.5%) perceived high involvement in the management of diabetes (Figure 1).

## Discussion

The primary goal of this study was to determine how much CPPs are actually involved in managing diabetes compared to how much they perceived to be involved. CPPs are well-known globally and play a variety of roles in public health, from primary dispensing to deeper participation in medical issues (Eades et al., 2011; Mossialos et al., 2015). Before taking any action, such as closely monitoring, offering customised intervention and support, and addressing the existing gaps, it is essential to assess their actual involvement in the management of diabetes. Evidence from throughout the globe shows that chemist intervention in diabetic patients led to a lower glucose level and a better prognosis (Farag Mohamed et al., 2021; Al Assaf et al., 2022; Malik et al., 2022; Pousinho et al., 2016). As a result, this study looked into CPPs’ current actual practice in diabetes management at CDROs in Northwest Ethiopia.

The current study showed discrepancies and lower involvement in providing services during actual practice, with an overall involvement of 17.8% as compared with a self-reported perceived level of involvement (73.5%) in managing diabetes. Most CPPs only provided basic traditional dispensing services like receipt of prescriptions, interpretation or evaluation of the prescriptions, and supply of the prescribed medication. The finding is consistent with earlier findings (Laliberté et al., 2012; Arif et al., 2023; Wang et al., 2021) that reported fewer participants actually involved in providing such activities. This may result from their limited knowledge and skills, which can result in their lower actual involvement in such healthcare services. In support of this evidence, most of the study participants in the self-reported study were diploma holders in their academic qualifications. CPPs with a higher education background might be more involved because they might have more recent information and communication skills for clients and the ability to engage in healthcare activities than CPPs with lower education levels (Sendekie et al., 2023). Additionally, individuals could easily acquire up-to-date information and apply the knowledge in practice, providing them with a better level of involvement in diabetes care. A survey of pharmacy students pursuing a bachelor's degree in the study environment regarding their participation in healthcare-related activities while attachments highlighted the significance of professional training, expertise, and setting standards for the services (Gelayee & Mekonnen, 2018). Increased workload, limited guidance resources, lack of counseling space, and insufficient management support could hinder their involvement (Nsengimana et al., 2022; Gelayee & Mekonnen, 2017; Dhippayom et al., 2013; Arif et al., 2023; Sendekie & Netere, 2022; Sia et al., 2020; El Hajj et al., 2016). Improved education, training, and addressing barriers likely emerge as solutions for increased involvement.

Despite the fact that CPPs were thought to be highly involved in a variety of public health services, including recommendations on how to reduce health risks like smoking, salt and alcohol consumption, weight management, blood pressure and blood glucose monitoring, medication adherence, prescription treatment, and general medication and health information, their actual practice fell short of their self-reported perception of how involved they were. This finding is consistent with earlier studies (Arif et al., 2023). This could be due to a lack of sufficient knowledge and skills and potential barriers related to the setting or the healthcare system. The findings suggest the need for training and improving educational backgrounds. They must not only improve their education but also their knowledge, skills, and confidence through training in diabetes management services. However, their lower actual involvement might also be because of a lack of commitment to providing such services. Surprisingly, in the current finding, almost all pharmacists did not request a prescription, despite the majority of them perceived to promote prescription medications in a self-reported study. The finding may emphasise the need to explore CPPs’ attitudes toward the public's healthcare priorities and their actual efforts to tackle their shortcomings to enhance their involvement in diabetes management. In such cases, the need for regular monitoring and control by regulatory bodies might be important. Pharmacists need to be highly vigilant to provide expected services in managing diabetes.

In this study, the actual involvement of pharmacists in the modification of lifestyles and avoiding risky behaviours, such as modification of diets including a cholesterol-free diet, physical activity, alcohol consumption, salt restriction, and smoking cessation, and counseling medication adherence to treatment and dosage regimens and possible side effects was limited compared with their perceived level of involvement. This can be due to a lack of training in diabetes management techniques among CPPs, which needs to be improved if CPPs are to provide efficient services. The study findings also showed that most of the participants didn’t receive on-the-job training in the management of diabetes. In order to improve, community health authorities, such as national chronic illness monitoring authorities, and other stakeholders should be frequently involved in monitoring CPPs. Collaborations between CDROs, healthcare authorities, and pertinent educational training institutes could be encouraged to include CPPs in programmes and workshops that promote public health priorities like diabetes.

Pharmacists’ actual involvement in counseling for routine monitoring of weight, blood pressure, and blood glucose monitoring and their actual involvement in the monitoring of weight, blood pressure, and blood glucose was limited as compared with their self-reported perceived level of involvement. Good foot care counseling, and over-the-counter and herbal drugs counseling services were also found to be very low in actual practice as compared with their perceived level of involvement. More importantly, extremely fewer pharmacists counsel the patient to consult physicians or general healthcare practitioners for further management. These findings are contrary with previous studies (Sia et al., 2020; Katoue et al., 2013). Differences in settings, with past studies in more supportive environments, could contribute to the observed gap in engagement. However, the findings were consistent with previous research on CPPs’ roles in the management of diabetes (Erku et al., 2017; Teka & Baye, 2018) and metabolic syndrome (Belachew et al., 2020). The findings may highlight the importance of training to monitor blood glucose and other parameters. In addition, pharmacists also need to be vigilant to provide professional activities.

Alongside robust training programmes, practice managers and policymakers can unleash the full potential of community pharmacists in the fight against diabetes, significantly improving patient outcomes and promoting a more accessible and comprehensive healthcare system. They need to collaborate in terms of resource allocation, creating inter-professional collaboration, developing targeted patient education programmes, and advocating for policy changes that expand the scope of practice for pharmacists in diabetes care and public health priorities.

In general, this study focused on the extent of CPPs’ actual practices in managing diabetes. The motivation for CPPs to offer diabetes care may come from the fact that promoting healthy behaviours among the general public is an essential population approach for reducing the burden of diabetes. Finally, the study suggests that future research focuses on pharmacists’ attitudes and beliefs, obstacles to providing effective practices, and challenges in the study settings using a qualitative study of their role in diabetes management.

### Study strengths and limitations

The current study produced a thorough conclusion about the extent of CPPs’ practices in the management of diabetes, which can advance the body of knowledge for healthcare professionals, patients, and other interested parties. Admittedly, this study does have some limitations. The results of this study may initially not apply to all CPPs across the nation, especially those in remote locations. The results may indicate the need for additional in-depth analysis using qualitative research, with a focus on identifying potential obstacles and contributing variables to optimal diabetes management by CPPs. Future studies that take into account rural settings are also advised by the study. To increases their involvement, further on-duty real monitoring and regular bodies’ participation may be necessary. Finally, the researchers expect that their findings will contribute to the body of knowledge already known on the subject and close a gap in the literature. It can also provide policymakers with information on how to enhance and incorporate CDROs into diabetes management practices and national initiatives that would aid in reducing the rising burden of diabetes on the nation.

### Conclusion and recommendations

The finding reveals a significant discrepancy between CPPs’ actual practices and their perceived level of involvement in managing diabetes. Most CPPs practice only basic activities like receipt of prescriptions and supply of medications, and expected services in terms of providing lifestyle modification, detailed medication-related information, and prevention strategies were not addressed, despite being perceived to be highly involved in a self-reported cross-sectional study. The findings may suggest pharmacists should be more vigilant and professional in managing patients with diabetes. Standardised guidelines focusing on community-based patient care could be important to enhance the actual practices of pharmacists on public health issues. In addition, a qualitative study focusing on the barriers to actual practices in future research may be necessary.

**Abbreviations**

CDROs, Community Drug Retail Outlets;
CPPs, Community pharmacy professionals.

## Supplementary Material

Supplemental Material

## Data Availability

All necessary materials are within the manuscript. The datasets generated and/or analysed during the current study is available upon reasonable request.
