# Supplementary material for: Actual practices of community pharmacists in the management of diabetes: a comparison of simulated patient-based study with perceived role of involvement
Source: J Pharm Policy Pract. 2024 Mar 28;17(1):2326381. doi: 10.1080/20523211.2024.2326381 (PMC10984231; doi:10.1080/20523211.2024.2326381)
Supplement: Supplemental Material [file JPPP_A_2326381_SM4482.docx]

**Self-reported involvement data collection form**

| Diabetes management activities | Level of CPPs’ response (n, %) | | | | |
| --- | --- | --- | --- | --- | --- |
|  | Not involved | Little involved | Uncertain | Involved | Very involved |
| **Medication review and counselling** | | | | | |
| Request prescription medication |  |  |  |  |  |
| Dosage regimen and detail possibles side effects |  |  |  |  |  |
| Adherence to treatment |  |  |  |  |  |
| Cautions of over-the-counter drugs or herbal products |  |  |  |  |  |
| **lifestyle and self-care modification related items** | | | | | |
| Salt restriction |  |  |  |  |  |
| Smoking cessation |  |  |  |  |  |
| Alcohol restriction |  |  |  |  |  |
| Exercise and physical activity |  |  |  |  |  |
| Weight reduction by non-weight bearing diet |  |  |  |  |  |
| Consumption of cholesterol free-diets |  |  |  |  |  |
| Consumption of vegetables |  |  |  |  |  |
| **Diabetes education and clinical intervention** | | | | | |
| Routine weight, blood pressure and blood glucose monitoring |  |  |  |  |  |
| Involving in measuring weight, blood pressure, and blood glucose |  |  |  |  |  |
| Good foot care techniques |  |  |  |  |  |
| Consult physicians/medical practitioners for further management |  |  |  |  |  |

**Actual practice data collection form**

| Practice items pharmacist could expect to counsel for a SP patient on: | Actual involvement | |
| --- | --- | --- |
|  | Yes | No |
| Request prescription medication |  |  |
| Salt restriction |  |  |
| Smoking cessation |  |  |
| Alcohol restriction |  |  |
| Adherence to treatment |  |  |
| Dosage regimen and detail possibles side effects |  |  |
| Exercise and physical activity |  |  |
| Routine weight, blood pressure and blood glucose monitoring |  |  |
| Weight reduction by non-weight bearing diet |  |  |
| Consumption of cholesterol free-diets |  |  |
| Consumption of vegetables |  |  |
| Involving in measuring weight, blood pressure, and blood glucose |  |  |
| Good foot care techniques |  |  |
| Cautions of over-the-counter drugs or herbal products |  |  |
| Consult physicians/medical practitioners for further management |  |  |
